# A contingency management approach for treatment of methamphetamine use disorder and human immunodeficiency virus antiretroviral treatment adherence in pregnancy to prevent mother-to-child transmission: a case report

**DOI:** 10.1186/s13256-022-03391-x

**Published:** 2022-04-27

**Authors:** Suzanne Turner, Maya Nader, Erin Lurie

**Affiliations:** 1 Department of Family Medicine, McMaster University, 100 Main St W 5th Floor, Hamilton, ON L8P 1H6 Canada; 2 St. Michael’s Hospital, Unity Health, 36 Queen St E, Toronto, ON M5B 1W8 Canada; 3 Department Family and Community Medicine, University of Toronto, 500 University Ave, Toronto, ON M5G 1V7 Canada

**Keywords:** Human immunodeficiency virus (HIV), Pregnancy, Substance use, Contingency management, Case report

## Abstract

**Introduction:**

This review highlights the rising prevalence of methamphetamine use in pregnancy in North American and the difficulty of managing active human immunodeficiency virus infection in a pregnant woman while actively using methamphetamines. Multidisciplinary medical teams with knowledge of addiction medicine, infectious disease management, and pregnancy are needed to provide combined expert care to reduce the harms associated with substance use and improve adherence to antiretroviral treatment. We report the case of a treatment-naïve pregnant patient with human immunodeficiency virus who was actively using methamphetamines. The patient was able to initiate and adhere to antiretroviral treatment while taking a prescription stimulant in a contingency management paradigm. To the best of our knowledge, this is the first documented case of prescription stimulants being used in pregnancy to improve adherence to antiretroviral medications.

**Case presentation:**

A 32-year-old white woman with untreated human immunodeficiency virus, a newly diagnosed pregnancy, and actively using methamphetamines presented to a drop-in combined prenatal care and addiction medicine clinic. After initiating a prescription amphetamine in a contingency management paradigm, she was adherent to human immunodeficiency antiretroviral treatment and had a fully suppressed viral load throughout the remainder of her pregnancy.

**Conclusion:**

Active treatment of methamphetamine use disorders with prescription stimulants, coupled with contingency management, may represent a mechanism to engage patients in care and improve adherence to antiretroviral treatment (and prevent mother-to-child-transmission of human immunodeficiency virus).

## Introduction

Methamphetamine use in North America is a rising concern, with methamphetamine-associated overdose rates increasing between 170% and 360% in some parts of Canada and hospitalizations related to methamphetamine-related harms rising between 500% and 800% [1]. Estimating methamphetamine exposure in pregnancy is challenging secondary to polysubstance use and the limitations of retrospective trials. In two California-based studies, the use of methamphetamines in pregnancy was estimated at between 0.4% and 0.7% [2, 3]. Methamphetamine use in pregnancy is associated with significant maternal and fetal effects. Spontaneous abortion, stillbirth, gestational hypertension, preeclampsia, preterm labor, and birth and placental abruption are all more common in methamphetamine-exposed pregnancies [4, 5]. Fetal effects include intrauterine growth restriction and low birth weight [6]. There are currently no approved pharmacological treatments for methamphetamine use disorder in pregnancy or outside of pregnancy [7]. Prescription amphetamines at high doses were associated with an increased chance of cocaine-negative urine and sustained self-reported abstinence among nonpregnant, stimulant users but did not change rates of treatment retention in at least one metaanalysis [8]. However, these results were not supported by an earlier Cochrane review [9]. Generally speaking, there is mixed and low-quality evidence to support or refute prescription psychostimulants for stimulant use disorder treatment.

Contingency management, the process by which positive behaviors (such as negative urine drug screens, attendance at medical appointments) are rewarded and negative behaviors result in consequences, is considered the gold-standard treatment for stimulant use disorders outside of pregnancy [10]. Contingency management programs have been shown to reduce illicit substance use in pregnancy and improve prenatal care attendance [11–16]. Financial rewards or gift cards are the most common type of reward associated with prenatal care attendance. Contingency management has been utilized in several studies to improve adherence to human immunodeficiency virus (HIV) treatment with antiretroviral therapy [17].

People who use drugs (PWUD) account for approximately 20% of HIV-positive Canadians and represent 30% of new HIV infections [18]. In nonpregnant populations, PWUD have been shown to start treatment later than other populations [19, 20]. Untreated HIV infection in pregnancy is associated with adverse outcomes such as an increased risk of miscarriage, stillbirth, intrauterine growth restriction, low birth weight, and chorioamnionitis [21]. Prevention of mother-to-child transmission is the focus of most antiretroviral treatment (ART) in pregnancy, with maximal viral load suppression being the goal as early in pregnancy as possible with sustained response resulting in the lowest vertical transmission rates [22, 23]. Identification of the barriers to sustained viral suppression is essential to the process of preventing mother-to-child transmission (MTCT) [23].

We report herein the case of a treatment-naïve HIV-positive patient who presented for prenatal care while actively using methamphetamines. A contingency management approach using a prescription stimulant was successful in helping initiate and maintain ART, which resulted in rapid and sustained virological suppression and subsequently prevented MTCT of HIV. This case report follows the CARE guidelines.

## Case presentation

The patient was a 32-year-old white woman who presented to a specialized combined care program offering obstetrical care and addiction medicine support in a single visit, at 6 weeks gestation in her first pregnancy. She reported using 200–300 Canadian dollars worth of crystal methamphetamine daily via a smoked route and was diagnosed with a stimulant use disorder. Complicating her presentation was a diagnosis of HIV made 2 years prior. She believes she contracted HIV through sexual activity with a known HIV-positive partner. The patient had never been on HIV treatment, and there were no recent viral loads, CD4 counts, or genotypes to guide treatment. She denied other substance use history (alcohol, cocaine, benzodiazepines, opioids including fentanyl and heroin, and cannabis). She did smoke 1 pack of cigarettes per day and had quit smoking after the diagnosis of pregnancy.

The patient’s psychiatric history included a childhood diagnosis of attention deficit hyperactivity disorder (ADHD). This was diagnosed at the age of 13, when she was started on treatment with extended-release methylphenidate. She continued this medication with significant improvement in her academic function. She continued extended-release methylphenidate until she was 20 years old and decided to stop taking it when she was in university, as she did not like how the medication made her feel and the perceived impacts it had on her personality. She denied any current or past symptoms of anxiety, depression, psychosis, obsessive compulsive disorder, or eating disorders. She had no judicial system involvement.

The patient reported that her main driver for methamphetamine use was reduction in her ADHD-type symptoms, particularly related to organization, concentration, and ability to engage in employment. On becoming pregnant, the patient was able to reduce her methamphetamine use from 200 to 300 Canadian dollars per day to 60 Canadian dollars per day. However, she had difficulty further reducing her use from this amount. She did not think she would be able to remember to take antiretroviral therapy on a regular basis without treatment of her ADHD symptoms and reduction in her methamphetamine use (which required her to spend a great deal of time during the day acquiring and using the substance).

The risks and benefits of stimulant treatment during pregnancy were reviewed with the patient. The patient provided formal consent to treatment and was aware this was an off-label treatment for stimulant use disorder and provided consent despite limited safety data for lisdexamfetamine in pregnancy.

A contingency management approach requiring the patient to attend the pharmacy daily for observed stimulant therapy was employed. This approach provided carried (take-home) doses of her stimulant for negative urine screens. Weekly urine drug screens were sent to a tertiary care hospital-based laboratory that was able to discern prescription stimulants from illicit methamphetamines. The patient was able to stop illicit methamphetamine use at 15 weeks’ gestation on a daily dose of lisdexamfetamine 30 mg. She obtained full carried doses (one observed dose and six carried doses 20 weeks after starting therapy). This was based on a carry schedule like local methadone programs, where a once weekly carried dose was granted for four weekly negative urine drug screens. Sunday carried doses were provided immediately secondary to limited pharmacy availability.

As the patient indicated that the daily observed lisdexamfetamine helped with her organization, she started on daily observed ART with a single tablet regimen containing emtricitabine, rilpivirine, and tenofovir. At initiation of ART, her viral load was 8530 and her CD4 count was 790. The patient’s viral load after 1 month of daily observed emtricitabine, rilpivirine, and tenofovir was undetectable.

She engaged in all weekly appointments for combined obstetrical and addictions care. She received all standard obstetrical care including blood work, immunizations, and ultrasounds. After 20 weeks on daily observed lisdexamfetamine, she was transitioned to six carries with once weekly observed dosing. Her second trimester, third trimester, and labor and delivery blood work revealed an undetectable viral load. Her urine drug screens continued to be consistent with self-reports of no illicit methamphetamine use.

The patient delivered a male child via spontaneous vaginal delivery at term and received more than 4 hours of zivovudine. His Apgar scores were 8 and 9, and his birthweight was 3255 g. He was followed by a tertiary care neonatal HIV clinic. He received 4 weeks of treatment with zidovudine. Following his newborn and 4-week blood work, he was taken off the antiretrovirals secondary to a negative viral load.

The patient was followed for 6 weeks postpartum. During this time, the patient continued lisdexamfetamine, and her urine drug screens remained negative in the postpartum period. She remained adherent to her ART.

## Discussion

This case demonstrates the complexity of managing a pregnant patient with a concurrent stimulant use disorder and untreated HIV. A multidisciplinary team with understanding of HIV treatment (in pregnancy), prenatal care, and advanced addiction medicine was needed to engage and retain this patient in care. At the core of this patient’s treatment experience was a drop-in clinic for pregnant patients with substance use disorders that employed a flexible, harm-reduction approach that supported the patient to attend despite ongoing substance use.

As methamphetamine use increases in North America and becomes more common in pregnancy [1–3], there will need to be ongoing investigation of pharmacotherapies to improve outcomes given the negative maternal and fetal effects of methamphetamines [4–6]. Given the lack of evidence to support pharmacotherapy for stimulant use disorders [8, 9], there is a need to aggressively treat concurrent conditions that could improve outcomes, such as the case of ADHD in this case. A recent study shows emerging evidence for mirtazapine in reducing stimulant use and HIV-risk behaviors in men who have sex with men [24] and in a recent review may be associated with small reductions in methamphetamine use in cis-gender men and transgender women [25]. This may represent an area for future research in other high-risk populations, including pregnant patients.

Contingency management not only is a powerful tool in substance use disorders but has also been shown to improve rates of prenatal care attendance [10–16]. In this case, the patient was highly motivated to stop using methamphetamine use, and therefore a contingency management approach using a treatment for her ADHD was reinforcing. As she gradually improved in her function, she returned to work and as a result, having carried doses of her prescription stimulant both rewarded her abstinence but also allowed her to engage in further activities of social and occupational functioning. In this case, the use of a prescription stimulant in a contingency paradigm also allowed the treatment team to track her initial adherence to her antiretroviral regimen as she took both the ART and the stimulant as witnessed doses. This case also highlights the need to identify and aggressively remedy barriers to ART in pregnancy to prevent MTCT of HIV.

## Conclusion

This case report describes the novel treatment of a methamphetamine use disorder with prescription stimulants, coupled with contingency management, and may represent a mechanism to engage patients in care and improve adherence to antiretroviral therapy in pregnancy. Rapid suppression of the patient’s viral load within the first month of treatment, followed by sustained virologic suppression, provided the patient with ongoing motivation to continue her ARVs. The combination of the psychostimulant tied to daily witnessed ARV treatment and then increasing rewards of take-home doses in a contingency paradigm provided a multipronged approach to the treatment of this patient that ultimately prevented mother-to-child transmission of HIV.

## Data Availability

Not applicable.

## References

[1] Canadian Centre for Substance Abuse (CCSA). Methamphetamine Use in Canada. Canadian Centre for Substance Abuse (CCSA); 2020. https://www.ccsa.ca/sites/default/files/2020-04/CCSA-Methamphetamine-Use-Harms-Canada-Infographic-2020-en.pdf. Accessed 20 Dec 2021.

[2] Vega WA, Kolody B, Hwang J, Noble A. Prevalence and magnitude of perinatal substance exposures in California. N Engl J Med. 1993;329(12):850–4. 10.1056/NEJM199309163291207. 8355744

[3] Gorman MC, Orme KS, Nguyen NT, Kent EJ 3rd, Caughey AB. Outcomes in pregnancies complicated by methamphetamine use. Am J Obstet Gynecol. 2014;211(4):429.e1-7. 10.1016/j.ajog.2014.06.005 (**Epub 2014 Jun 4**). 24905417

[4] Committee Opinion No. 479: Methamphetamine abuse in women of reproductive age. Obstet Gynecol. 2011;117(3):751–5. 10.1097/AOG.0b013e318214784e. 21343793

[5] Brecht ML, Herbeck DM. Pregnancy and fetal loss reported by methamphetamine-using women. Subst Abuse. 2014;8:25–33. 10.4137/SART.S14125. PMC4011724 24855369

[6] Sankaran D, Lakshminrusimha S, Manja V. Methamphetamine: burden, mechanism and impact on pregnancy, the fetus, and newborn. J Perinatol. 2021. 10.1038/s41372-021-01271-8 (**Epub ahead of print**). PMC13237812 34785765

[7] Smid MC, Metz TD, Gordon AJ. Stimulant use in pregnancy: an under-recognized epidemic among pregnant women. Clin Obstet Gynecol. 2019;62(1):168–84. 10.1097/GRF.0000000000000418. PMC6438363 30601144

[8] Tardelli VS, Bisaga A, Arcadepani FB, Gerra G, Levin FR, Fidalgo TM. Prescription psychostimulants for the treatment of stimulant use disorder: a systematic review and meta-analysis. Psychopharmacology. 2020;237(8):2233–55. 10.1007/s00213-020-05563-3 32601988

[9] Pérez-Mañá C, Castells X, Torrens M, Capellà D, Farre M. Efficacy of psychostimulant drugs for amphetamine abuse or dependence. Cochrane Database Syst Rev. 2013;2(9):CD009695. 10.1002/14651858.CD009695.pub2 PMC11521360 23996457

[10] Prendergast M, Podus D, Finney J, Greenwell L, Roll J. Contingency management for treatment of substance use disorders: a meta-analysis. Addiction. 2006;101(11):1546–60. 10.1111/j.1360-0443.2006.01581.x 17034434

[11] Elk R, Mangus L, Rhoades H, Andres R. Grabowski J. Addict Behav. 1998;23(1):57–64. 10.1016/s0306-4603(97)00020-8 9468743

[12] Jones HE, Haug N, Silverman K, Stitzer M. Svikis D. Drug Alcohol Depend. 2001;61(3):297–306. 10.1016/s0376-8716(00)00152-6 11164694

[13] Petry NM, Martin B. Prize reinforcement contingency management for cocaine dependence: integration with group therapy in a methadone clinic. Simcic F Jr J Consult Clin Psychol. 2005;73(2):354–9. 10.1037/0022-006X.73.2.354 15796645

[14] Chang G, Carroll KM, Behr HM, Kosten TR. Improving treatment outcome in pregnant opiate-dependent women. J Subst Abuse Treat. 1992;9(4):327–30. 10.1016/0740-5472(92)90026-k PMC3677856 1479630

[15] Hand DJ, Ellis JD, Carr MM, Abatemarco DJ, Ledgerwood DM. Contingency management interventions for tobacco and other substance use disorders in pregnancy. Psychol Addict Behav. 2017;31(8):907–21. 10.1037/adb0000291. PMC5714659 28639813

[16] Carroll KM, Chang G, Behr H, Clinton B, Kosten TR. Improving treatment outcome in pregnant, methadone-maintained women: results from a randomized clinical trial. Am J Addict. 1995;4(1):56–9.

[17] Haug NA, Sorensen JL. Contingency management interventions for HIV-related behaviors. Curr HIV/AIDS Rep. 2006;3(4):154–9. 10.1007/s11904-006-0010-5 17032574

[18] The Canadian Aids Society. Injection Drug Users. The Canadian Aids Society; 2020. https://www.cdnaids.ca/resources/populations/people-use-drugs/. Accessed 20 Dec 2021.

[19] Suárez-García I, Sobrino-Vegas P, Dalmau D, Rubio R, Iribarren JA, Blanco JR, Gutierrez F, Montero Alonso M, Bernal E, García VD, Del Amo J, Cohort of the Spanish HIV Research Network (CoRIS). Clinical outcomes of patients infected with HIV through use of injected drugs compared to patients infected through sexual transmission: late presentation, delayed anti-retroviral treatment and higher mortality. Addiction. 2016;111(7):1235–45. 10.1111/add.13348 (**Epub 2016 Apr 14**). 26890155

[20] CASCADE (Concerted Action on SeroConversion to AIDS and Death in Europe) Collaboration. Changes in the uptake of antiretroviral therapy and survival in people with known duration of HIV infection in Europe: results from CASCADE. HIV Med. 2000;1(4):224–31. 10.1046/j.1468-1293.2000.00033.x 11737353

[21] Bernstein HB, Wegman AD. HIV infection: antepartum treatment and management. Clin Obstet Gynecol. 2018;61:122–36. 10.1097/GRF.0000000000000330 29309283

[22] Camacho-Gonzalez AF, Kingbo MH, Boylan A, Eckard AR, Chahroudi A, Chakraborty R. Missed opportunities for prevention of mother-to-child transmission in the United States. AIDS. 2015;29(12):1511–5. 10.1097/QAD.0000000000000710 PMC4502985 26244391

[23] Jean J, Coll A, Monda M, Potter JN, Jones D. Perspectives on safer conception practices and preconception counseling among women living with HIV. Health Care Women Int. 2016;37:1096. 10.1080/07399332.2015.1107068 PMC4841733 26492078

[24] Coffin PO, Santos GM, Hern J, Vittinghoff E, Walker JE, Matheson T, Santos D, Colfax G, Batki SL. Effects of mirtazapine for methamphetamine use disorder among cisgender men and transgender women who have sex with men: a placebo-controlled randomized clinical trial. JAMA Psychiat. 2020;77(3):246–55. 10.1001/jamapsychiatry.2019.3655. PMC6990973 31825466

[25] Naji L, Dennis B, Rosic T, Wiercioch W, Paul J, Worster A, Thabane L, Samaan Z. Mirtazapine for the treatment of amphetamine and methamphetamine use disorder: a systematic review and meta-analysis. Drug Alcohol Depend. 2022;232:109295. 10.1016/j.drugalcdep.2022.109295 35066460

